# COVID-19 related change in breast cancer diagnosis, stage, treatment, and case volume: 2019–2021

**DOI:** 10.1007/s10549-023-06962-8

**Published:** 2023-08-16

**Authors:** Judith A. Malmgren, Boya Guo, Mary K. Atwood, Paula Hallam, Laura A. Roberts, Henry G. Kaplan

**Affiliations:** 1 HealthStat Consulting, Inc, 12025 9th Ave NW, Seattle, WA 98177 USA; 2 School of Public Health, University of Washington, Seattle, WA USA; 3 Swedish Cancer Institute, 1221 Madison St, Seattle, WA 98104 USA

**Keywords:** COVID-19, Mammography, Breast cancer, Diagnosis, Stage, Treatment

## Abstract

**Purpose:**

Evaluate the COVID-19 pandemic impact on breast cancer detection method, stage and treatment before, during and after health care restrictions.

**Methods:**

In a retrospective tertiary cancer care center cohort, first primary breast cancer (BC) patients, years 2019–2021, were reviewed (n = 1787). Chi-square statistical comparisons of detection method (patient (PtD)/mammography (MamD), Stage (0-IV) and treatment by pre-pandemic time 1: 2019 + Q1 2020; peak-pandemic time 2: Q2-Q4 2020; pandemic time 3: Q1-Q4 2021 (Q = quarter) periods and logistic regression for odds ratios were used.

**Results:**

BC case volume decreased 22% in 2020 (N = 533) (p = .001). MamD declined from 64% pre-pandemic to 58% peak-pandemic, and increased to 71% in 2021 (p < .001). PtD increased from 30 to 36% peak-pandemic and declined to 25% in 2021 (p < .001). Diagnosis of Stage 0/I BC declined peak-pandemic when screening mammography was curtailed due to lock-down mandates but rebounded above pre-pandemic levels in 2021. In adjusted regression, peak-pandemic stage 0/I BC diagnosis decreased 24% (OR = 0.76, 95% CI: 0.60, 0.96, p = .021) and increased 34% in 2021 (OR = 1.34, 95% CI: 1.06, 1.70, p = .014). Peak-pandemic neoadjuvant therapy increased from 33 to 38% (p < .001), primarily for surgical delay cases.

**Conclusions:**

The COVID-19 pandemic restricted health-care access, reduced mammography screening and created surgical delays. During the peak-pandemic time, due to restricted or no access to mammography screening, we observed a decrease in stage 0/I BC by number and proportion. Continued low case numbers represent a need to re-establish screening behavior and staffing.

## Introduction

The first reported case of 2019-nCoV infection in the United States was in Washington State on January 19, 2020 and on March 23rd 2020, a statewide stay-at-home order was announced to remain in effect until May 30, 2020 and then lifted in a move to phased reopening [1, 2]. On March 19, 2020 statewide non-urgent procedures were prohibited in hospitals and ambulatory surgical facilities until May 18, 2020 [3]. Physician groups recommended postponement of breast cancer screening and some recommended delays set by a priority system [4, 5]. Most health care systems including ours started reopening to screening in the summer of 2020 after stay-at-home orders were lifted in May 2020 [3, 6].

In Washington State and in particular at our institution restrictions to movement, access to care and allowed procedures began to ease in June 2020 and stayed the same to the end of that year. With rapid uptake of COVID-19 vaccination in January 2021 for all health care workers, access to health care returned to more normal levels in 2021. Washington State and in particular the Puget Sound Region may have had more rapid uptake of vaccinations and ability to open to care than other parts of the country.

On December 11, 2020, the Federal Drug Administration issued an Emergency Use Authorization for the Pfizer-BioNTech COVID-19 vaccine and for the Moderna COVID-19 vaccine on December 18, 2020. [7] Soon after vaccinations of essential workers in hospitals and clinics began. [8] As vaccination coverage and eligibility increased in 2021, vaccines became more readily available. Health care became more accessible by the first quarter (Q1) of 2021 and returned to more normal levels in 2021, the second year of the pandemic.

The COVID-19 pandemic created an unprecedented interruption in health care access. Our objective is to conduct a comprehensive evaluation of real-world experience at a tertiary cancer care center, evaluating pandemic restrictions impact on mammography screening, method of detection, stage at diagnosis, treatment and reasons for delays during 2020 and into 2021 when pandemic restrictions eased, including the relationship between these outcomes.

## Methods

For our retrospective cohort study, we used patient data from our institutions’ breast cancer research registry database in the year prior to, during and after the pandemic restricted care delivery, 2019–2021. The database contains detailed information on method of diagnosis, patient characteristics, and stage at diagnosis. Clinical presentation characteristics including age, race, American Joint Committee Council on Cancer anatomic TNM stage, and method of detection by patient, mammography or other method were chart abstracted at time of diagnosis [9]. We included all women presenting with first primary breast cancer, biopsy confirmed and diagnosed in years 2019 to 2021 (N = 1797). Ten cases were dropped due to incomplete data (N = 1787). First primary breast cancer in the context of our study is when the patient has had no previous cancer diagnosis of any kind. We followed the STROBE observational study reporting guideline.

Incident BC cases were entered at time of diagnosis into the HIPAA compliant and IRB approved registry [IRB Study ID SWD39425-03]. This study was issued exemption status and approval by the Swedish Cancer Institute IRB program as the data is de-identified for analytic and study purposes. Information on each case demographics, diagnosis method, stage of disease, and treatment were included in initial data entry and as they occurred over time. Swedish Breast Care network radiology department utilization data was included in the IRB approval. All data used for analysis were de-identified at time of download prior to analysis.

At our institution, the dedicated Breast Cancer Research Registry Database has continuously entered BC patient information since 1995 by certified cancer registrars. The data is entered prospectively on all BC patients diagnosed or treated at our institution. Breast cancer detection method is a single variable in the registry with an option for detection method: patient (PtD), mammography (MamD), or other. Annual review of data integrity is conducted by the research staff.

Breast cancer detection method was obtained by medical record review at the time of diagnosis by a certified cancer registrar for each patient using the electronic medical chart and then input into the dedicated breast cancer research registry database. Mammography-detected breast cancer is disease discovered by routine mammography in the absence of complaints or known physical findings. Patient-detection was assigned if the patient detected breast symptoms, such as a palpable lump, pain, swelling, discharge, or bleeding prompting a clinic visit. Patients with self-detected tumors may have subsequent diagnostic mammograms or ultrasound done but are still labeled as a patient-detected breast cancer. ‘Other’ breast cancer detection includes physician detected or incidental findings from non-screening imaging for other complaints. If the detection method was ambiguous or incomplete, the tumor detection method was marked as unknown and those cases were excluded from the analysis. Diagnosis date for mammography detected BC is assigned on the date of positive biopsy after the BC is detected by mammography or confirmed by a diagnostic mammogram and ultrasound.

The institutional radiology department has billing utilization data from four imaging sites in the Swedish Breast Care network. Mammography utilization records are separated into screening and diagnostic imaging mammograms. Three of the four sites were temporarily closed on March 23, 2020 during the initial phase of the pandemic [3]. No mammography screening was conducted in April 2020 but diagnostic imaging for presenting non-screen detected cases continued. Restrictions on non-urgent procedures were reduced and mammography screening was resumed on a limited basis May 18, 2020 [3]. The utilization data is not linked to the Breast Cancer Registry Database used for this analysis but is at the same institution.

**Fig. 1:**
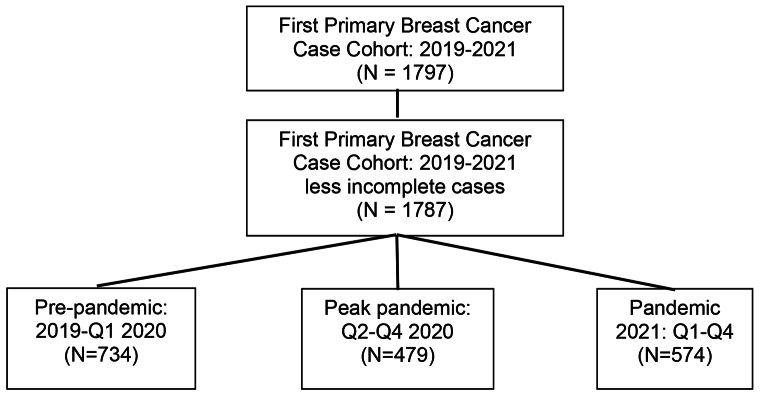
Consort Diagram

In our cohort study of breast cancer detection in the year prior to, during and after pandemic restricted care delivery, 2019–2021, we set the time periods to correlate with times of restricted access to health care. Pre-pandemic time 1 was all of 2019 and the first quarter (Q1) of 2020 before the State of Emergency Stay at Home orders were issued in Washington State effective March 23, 2020 [10]. Peak pandemic time 2 was the second to fourth quarter of 2020 when the nadir of screening and case diagnoses occurred. Pandemic 2021 time 3 was Q1-Q4 2021 after movement restrictions were lifted, protections were in place and vaccinations had begun. (Consort diagram Fig. 1)

Statistical analyses were done using de-identified data. IBM SPSS Statistics version 28 was used for all statistical analysis [11]. Statistical comparisons were done using the three time periods, (1) pre-pandemic time: 2019-Q1 2020, (2) peak-pandemic time: Q2-Q4 2020 and (3) pandemic 2021: Q1-Q4 2021 (Q = quarter). Pearson chi- square tests were used for bivariate analysis of dichotomous variables and analysis of variance was used for mean comparisons. All p values are two tailed with significance at the 0.05 level. Fishers exact testing was used in cases when a cell had less than 5 cases.

An adjusted binomial logistic regression model was used to calculate odds ratios and 95% confidence intervals for probability of outcome anatomic TNM stage 0/I. The model construction was informed by variables significant in the Pearson chi-square analyses (Table 1) including the three time periods pre-pandemic, peak pandemic and pandemic 2021. We are testing if the outcome of decreased stage 0/I is related to restricted mammography screening that was a non-essential service under Washington State mandates during the peak-pandemic time: Q2-Q4 2020. [12, 13]

**Table 1:** Chi-square analysis by COVID-19 pandemic diagnosis time periods: 2019–2021 (N = 1787)

	Pre-pandemic 2019-Q1 2020 (N = 734)	Peak Pandemic Q2-Q4 2020 (N = 479)	Pandemic 2021 (N = 574)	
	No. (%)	No. (%)	No. (%)	p value
Age				
<65	493 (67%)	321 (67%)	380 (66%)	0.929
>=65	241 (33%)	158 (33%)	194 (34%)	
Race				
White	519 (71%)	336 (70%)	382 (67%)	0.237
Non-white	215 (29%)	143 (30%)	192 (33%)	
How Detected				
Mammography	466 (64%)	278 (58%)	406 (71%)	< 0.001
Patient	221 (30%)	171 (36%)	143 (25%)	
Other	47 (6%)	30 (6%)	25 (4%)	
TNM Anatomic Stage*				
0	157 (21%)	78 (16%)	128 (22%)	< 0.001
I	291 (40%)	181 (38%)	260 (45%)	
II	207 (28%)	157 (33%)	136 (24%)	
III	67 (9%)	42 (9%)	38 (7%)	
IV	12 (2%)	21 (4%)	12 (2%)	
Hormone Receptor (HR)				
HR+	643 (89%)	417 (89%)	494 (89%)	0.938
HR-	77 (11%)	52 (11%)	63 (11%)	
Hormone Therapy				
Yes	537 (73%)	367 (77%)	405 (71%)	0.086
No	197 (27%)	112 (23%)	169 (29%)	
Neoadjuvant therapy				
Yes	99 (13%)	120 (26%)	86 (16%)	< 0.001
No	634 (87%)	345 (74%)	465 (84%)	
Chemotherapy				
Yes	222 (30%)	171 (36%)	166 (29%)	0.047
No	511 (70%)	306 (64%)	403 (71%)	

*AJCC 8 anatomic stage

## Results

**Fig. 2:**
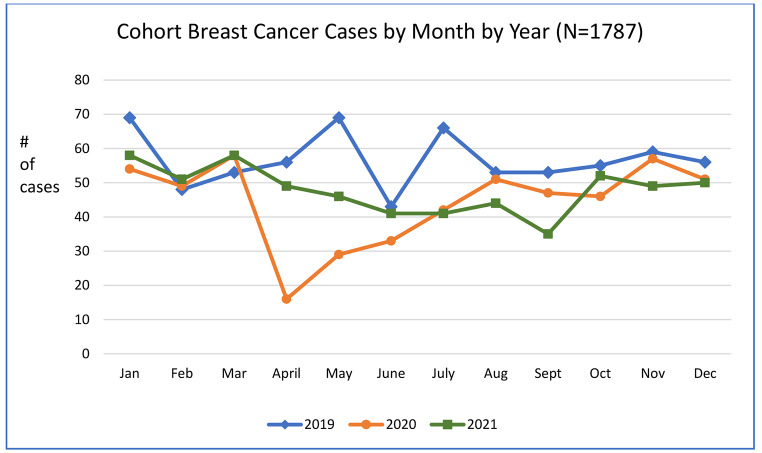
Cohort Breast Cancer Cases by Month by Year: 2019–2021 (n = 1787)

From 2019 to 2021, 1787 breast cancer cases were diagnosed at our institution. Compared to 2019 levels (N = 680), BC case volume was 22% lower in 2020 (N = 533) (p = .001) and 16% lower in 2021 (N = 574) (p = .012). (Fig. 2) Pre-pandemic case volumes did not differ from 2017 to 2019 [2017: n = 683, 2018: n = 687, 2019: n = 680].

**Fig. 3:**
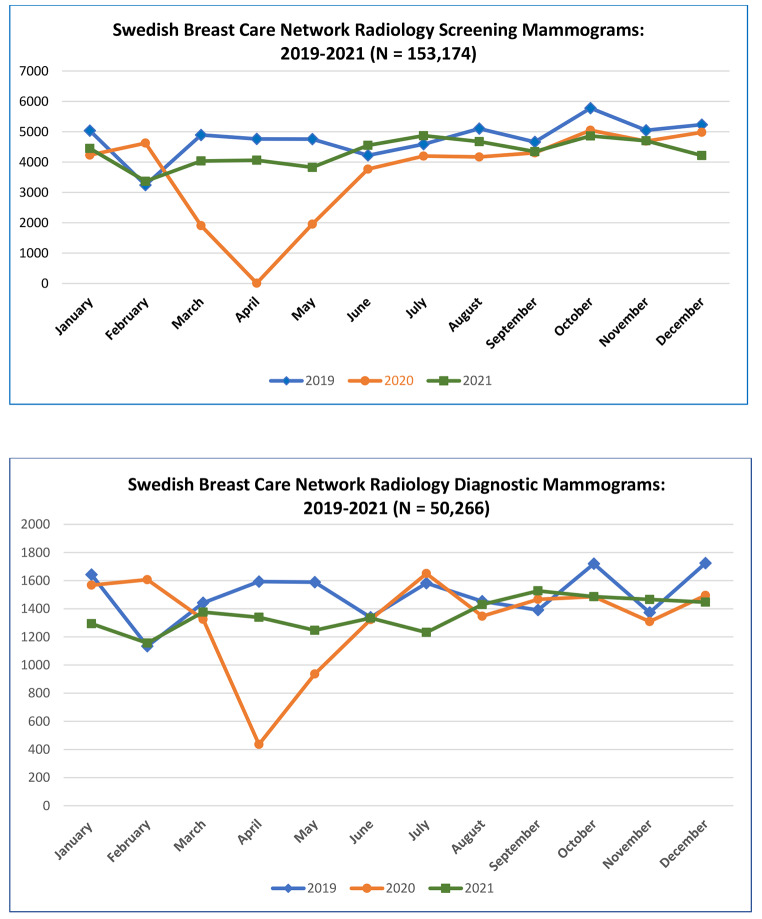
Swedish Breast Care Network Radiology Screening Mammograms (N = 153,174) and Diagnostic Mammograms (N = 50,266): 2019–2021

**Fig. 4:**
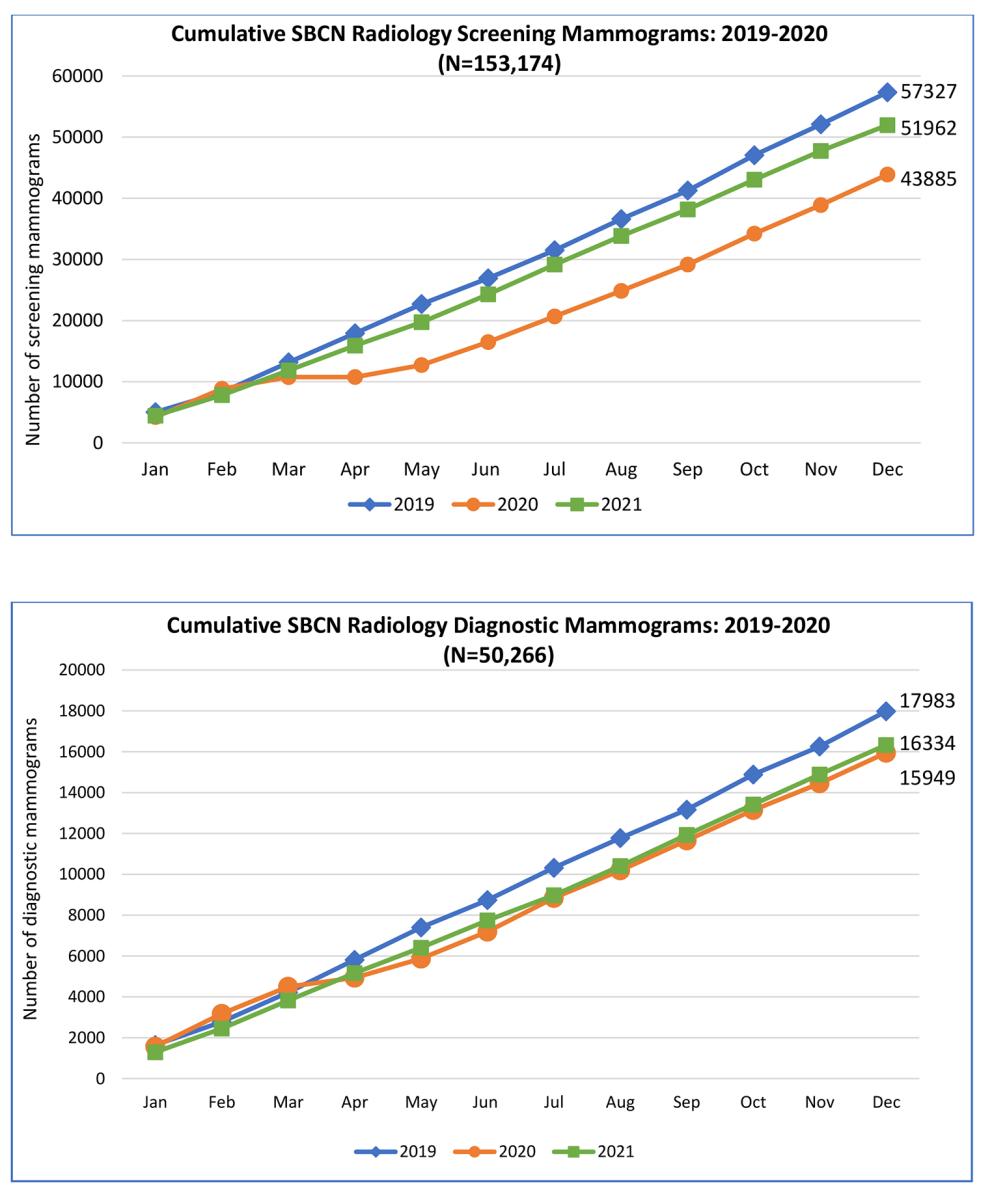
Swedish Breast Care Network Radiology (SBCN) Cumulative Screening and Diagnostic Mammograms 2019–2021

From institutional radiology department utilization data, breast cancer case volume maximum decline was reached in April 2020 when mammography screening was suspended during the time of restricted access and cessation of non-urgent procedures in the second quarter of 2020. Diagnostic mammography continued to be done on a case-by-case basis. (Fig. 3) Screening mammograms declined 23% in 2020 (n = 43,885) compared to 2019 (n = 57,327) and increased 15% in 2021 from 2020 levels (n = 51,962) but not up to 2019 case volumes. From the same radiology utilization data, diagnostic mammograms declined 11% in 2020 and increased in 2021 but not up to 2019 levels [2019: n = 17,983, 2020: n = 15,949, 2021: n = 16,334]. (Fig. 4)

**Fig. 5:**
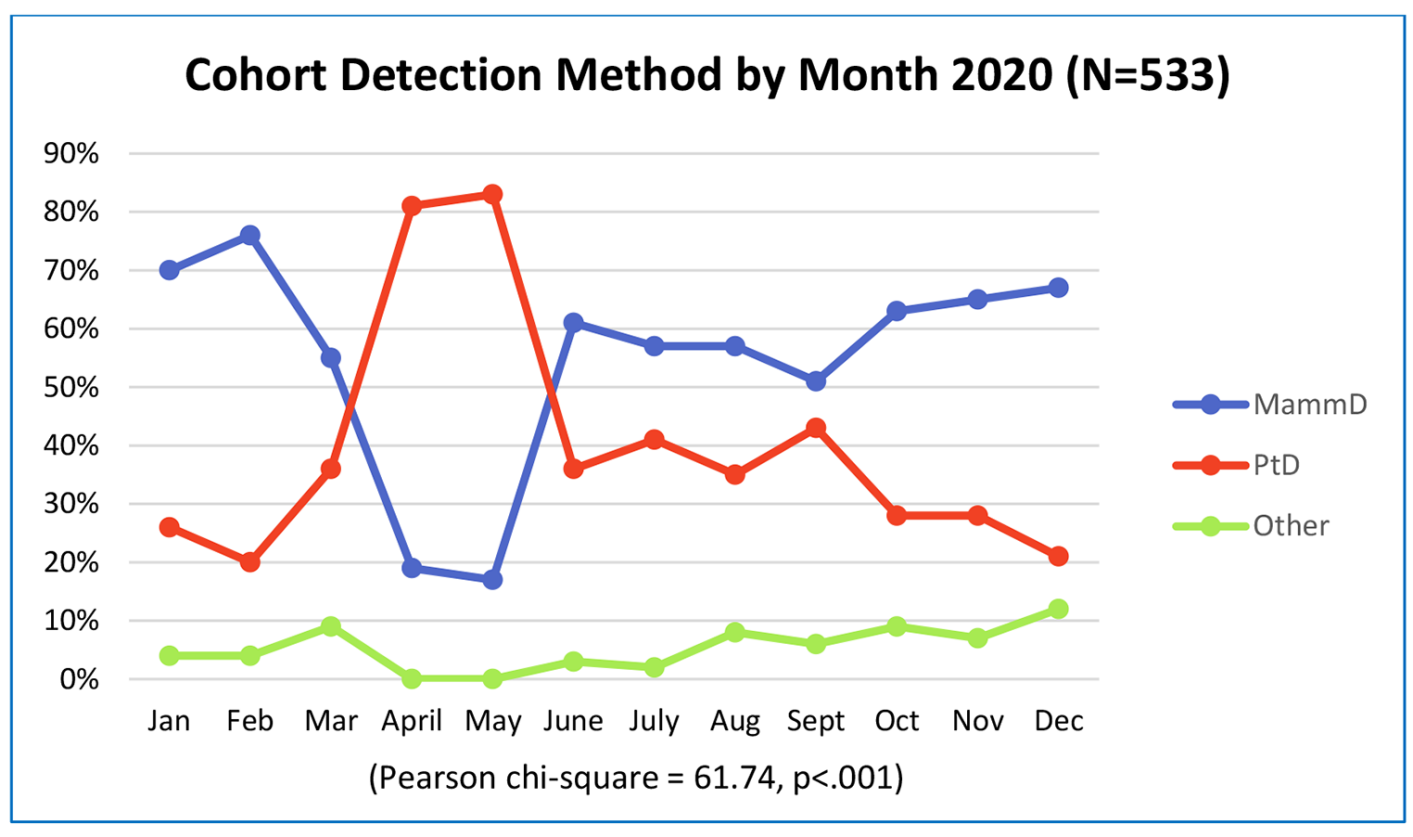
Cohort Change in Detection Method by Month: 2020 (N = 533)

In our breast cancer cohort data analysis, age at diagnosis and race did not differ over the three time periods. Detection method shifted with mammography-detected breast cancer cases dropping in peak pandemic: Q2-Q4 2020 from 64 to 58% and patient-detected breast cancer increasing from 30 to 36% (p < .001) [pre-pandemic: MamD BC (n = 466), peak pandemic: MamD BC (n = 278); pre-pandemic: PtD BC (n = 221), peak pandemic: PtD BC (n = 171)]. In the cohort analysis, the largest change was in April and May 2020 (p < .001). (Fig. 5) Mammography detected cases with biopsy assigned diagnosis dates in April and May 2020 put them in months when no screening mammograms were done.

**Fig. 6:**
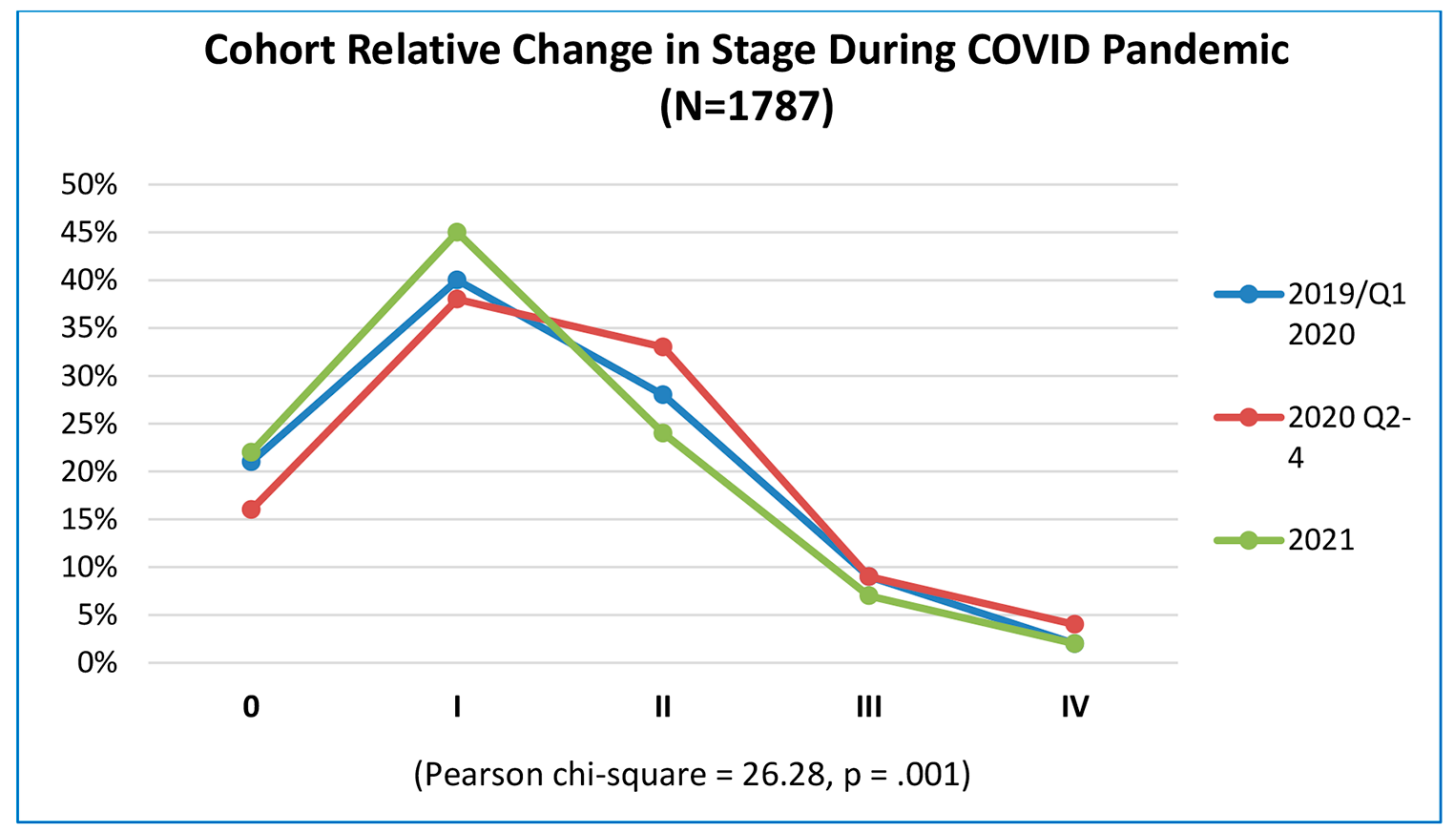
Cohort Relative Change in Stage during COVID: pre-pandemic: 2019/Q1 2020, peak pandemic: Q2-Q4 2020, pandemic: 2021

In pandemic time 2021 mammography detected breast cancer rebounded to 71%, 7% above the level observed prior to the pandemic [pre-pandemic: MamD BC = 64% (n = 466), pandemic 2021: MamD BC: 71% (n = 406) (p < .001)]. (Table 1) Concurrently, anatomic stage shifted with a drop in stage 0/I from pre-pandemic to peak-pandemic [pre-pandemic stage 0: 21% (n = 157), peak-pandemic stage 0: 16% (n = 78)] and stage I [pre-pandemic stage I: 40% (n = 291), peak-pandemic stage I: 38% (n = 181) (p < .001)]. Conversely in peak-pandemic, there was a relative higher proportion stage II and IV cancer from 28 to 33% stage II [pre-pandemic: n = 207, peak-pandemic: n = 157 (p < .001)], stage III no change and 2 to 4% stage IV [pre-pandemic: n = 12, peak-pandemic: n = 21 (p < .001)]. In pandemic time 2021, relative percent stage 0 increased to 22% of total (n = 128) and stage I to 45% of total (n = 260), but at overall case volume levels still below pre-pandemic time for each stage. In pandemic time 2021 stage II, III and IV percentages were below pre-pandemic levels [stage II 24% (n = 136), stage III 7% (n = 38), stage IV 2% (n = 12) (p < .001)]. (Fig. 6)

Hormone receptor status of diagnosed breast cancer cases did not change over time. Percent hormone therapy increased in peak-pandemic time but not significantly or numerically [pre-pandemic: n = 537 (73%), peak-pandemic: n = 367 (77%), p = .084)]. Neoadjuvant therapy, both chemo and hormone, increased from 13% (n = 99) pre-pandemic to 26% (n = 120) peak-pandemic and decreased to 16% in pandemic 2021 (n = 86) (p < .001). Twenty-three of the peak pandemic neoadjuvant therapy patients were given neoadjuvant hormone therapy due to COVID-19 related surgery delay (recorded in the chart). Chemotherapy treatment not including neoadjuvant therapy increased from 30% pre-pandemic (n = 222) to 36% peak-pandemic (n = 171) and back to pre-pandemic relative percentage in pandemic 2021 (29%, n = 166) (p = .047). (Table 1)

In a logistic regression model, outcome = stage 0/I BC, adjusted for age < 65/65+, race white/non-white and hormone receptor status present/absent, the odds of stage 0/I breast cancer diagnosis decreased 24% in the peak-pandemic time compared to pre-pandemic time [peak-pandemic: stage 0/I OR = 0.76, 95% CI: 0.60, 0.96, p = .021; age 65+: OR 1.46, 95% CI 1.18, 1.80, p < .001; race = white: OR = 0.85, 95% CI 0.69,1.05, p = .138; HR positive: OR = 2.53, 95% CI 1.86, 3.45, p < .001]. In the same model, pandemic 2021 odds of lower stage breast cancer diagnosis increased 34% compared to pre-pandemic [OR = 1.34, 95% CI: 1.06, 1.70, p = .014]. (Table 2)

**Table 2:** Logistic regression odds ratios: outcome = TNM stage 0-I: 2019–2021 (n = 1787)

	Odds Ratio	95% CI	p-value
Race			
White	reference		
Non-white	0.85	0.69, 1.05	0.138
Age			
Age 65+	reference		
Age < 65	1.46	1.18, 1.80	< 0.001
Hormone receptor status			
positive	reference		
negative	2.53	1.86, 3.45	< 0.001
Diagnosis time period			
Pre-pandemic: 2019-Q1 2020	reference		< 0.001
Peak pandemic: Q2-Q4 2020	0.76	0.60, 0.96	0.021
Pandemic 2021: Q1-Q4 2021	1.34	1.06, 1.70	0.014

## Discussion

During the COVID-19 pandemic, health screening and care access were limited to varying degrees with treatment and diagnosis delays depending on local regulation and jurisdiction of health care delivery. In our study of the pandemic impact at our institution in Washington State, we reviewed how breast cancer diagnosis methods, stage at diagnosis, treatment and case volumes were affected by this unprecedented natural disaster-like event and the resulting regulations put in place to curtail COVID-19 spread. We observed a cascade of effects from pandemic related restrictions that affected screening, stage at diagnosis, case volumes, and treatment delays with alternative therapy options enacted in 2020.

In the second quarter of 2020 the impact of COVID-19 closures and restrictions began to take effect at our institution and there was a decrease in breast cancer case volume. In April 2020, mammography screening was stopped while diagnostic mammograms continued on a limited basis dictated by case prioritization directives. During the peak pandemic time, Q2-Q4 of 2020, case volume declined and there was a numeric and proportional decrease in stage 0/I BC relative to stage II-IV BC due to the discontinuation and restriction of non-essential screening in Washington State during the peak pandemic period. In 2021 when mammography screening became more available, the trend reversed to more stage 0 and I, mammography detected breast cancer diagnosed but number of cases were still below 2019 pre-pandemic levels. Neoadjuvant therapy increased during peak-pandemic Q2-Q4 2020 when pandemic restrictions limited care access causing surgery delays and alternative therapies were made available. The most common COVID-19 related alternative treatment observed was pre-operative hormone therapy given to lower stage hormone receptor positive cases due to restrictions on non-urgent surgery. Other treatments affected or delayed were chemotherapy, radiation therapy and treatment planning.

Higher stage II-IV breast cancer diagnosis did not change significantly during the pandemic with a relative proportional increase due to declining number of mammography detected cases stage 0 and I in the peak pandemic time Q2-Q4 2020. In 2021, the relative proportion of stage II-IV BC declined when mammography screening was available and number of stage 0 and I breast cancers increased. In a comparison of only stage II-IV breast cancers, number and proportion of stage II-IV BC did not change significantly over the 3 time periods (data not presented).

Some preliminary studies of the effects of COVID-19 pandemic restrictions on health care access and/or screening for breast cancer have been reported. Decreased mammography screening occurred during the pandemic onset in 2020 [14–17]. Two studies reported higher proportion of higher stage or symptomatic disease in the 2020 shelter-in-place time period [18, 19]. One study reported increased neoadjuvant therapy [20].

Decline in diagnosed breast cancer case volumes was observed in the US in association with the COVID-19 pandemic in 2020 [21]. In Turkey, a study of a single institution found the volume of breast surgery declined in 2020 [22]. Velazquez et al. tracked screening mammograms at their institution during the 2019–2020 time period and observed a sharp decline in screening mammograms beginning in March 2020 with an increasing number of missed appointments and a slow increase later in 2020 but the number of screening mammograms remained below 2019 baseline levels [14]. In a large study of mammography utilization through 2021, a 40% decrease in mammography was observed nationally in 2020, with rates remaining below normal into 2021, but screening and diagnostic mammography were not differentiated [23]. Similarly, Doan et al. in a study of Medicare enrollee utilization of breast cancer screening found profound decreases in screening starting in March 2020 with continued monthly rate decreases related to increases in national COVID-19 infection rates [24]. Decreases in Medicare cancer screening observed did not resolve after initial pandemic surges.

In the Flanders region of Belgium, breast cancer screening invitation coverage dropped 10% in 2020 but the backlog of invitations was largely resolved in 2021 [25]. The authors concluded a minimal influence on willingness to screen existed but coverage of screening may have been impacted. Duffy et al. assessed the impact of the temporary COVID-19-related cessation of population screening on breast cancer deaths in England. They projected between 148 and 687 additional breast cancer deaths could occur depending on how quickly delays were caught up [26].

In 2020, Guth et al. observed fewer mammography detected breast cancers, more breast cancer detected by self-exam which were palpable on presentation, and fewer DCIS cases between 4/1/2020 and 3/31/2021 [27]. During the time when only essential surgery was allowed, more patients were treated with neoadjuvant chemotherapy and neoadjuvant hormonal therapy. They did not observe an increase in higher stage III and IV breast cancer and observed a decrease in radiation therapy. Zhou et al. observed a significantly lower number of stage I BC and higher amount of stage IV in 2020 after the start of the pandemic [28]. Trojanowski et al. observed a stage shift in 2021 with stage II BC more frequent than stage I and a significant increase in stage III [29].

On April 1, 2020, The American Society of Breast Surgeons issued a guide for use of neoadjuvant endocrine therapy related to surgical delays during the pandemic to assist with triaging breast cancer patients [30]. In 2020, authors Marti et al. and Thompson et al. discussed priorities and options for estrogen receptor positive breast cancer management due to surgical delays [31, 32]. Tonneson et al. observed a 10% increase in neoadjuvant endocrine therapy during the COVID-19 restricted access to care [33]. Habbous et al. observed a 20% increase in neoadjuvant systemic therapy during COVID-19-related care access, both hormonal and chemotherapy [34].

In our cohort, the percentage mammography detected breast cancer cases rebounded to above 2019 levels with a shift to earlier stage cancers very quickly in 2021, although actual number of cases remained below 2019 levels. These findings indicate the impact from delayed screening may have had minimal effect on compliant screeners who quickly made up their missed appointments. The continued lower than usual case volume of both screening mammograms and diagnosed BC cases overall indicate a missing portion of the potential screened population exists.

The 24% decrease in early stage 0 and I breast cancer in peak-pandemic time Q2-Q4 2020 was related to stoppage of mammography screening and limited access in 2020 followed by catch up screening in 2021 with early stage 0 and I breast cancer 34% higher than pre-pandemic year 2019/Q1 2020. However, mammography screening in the United States relies on opportunistic or invitational mammography screening depending on where one receives care, whether the care delivery organization has a reminder system, and type of insurance if one has health insurance. Some guidelines are based on United States Preventive Services Task Force, the American Cancer Society and other organizations recommendations unlike countries with singular organized screening programs [35–38]. Our institutional radiology department uses American College of Radiology (ACR) guidelines which recommend annual screening starting at age 40 [39]. Screening is therefore predicated on self-initiation of screening mammography or prompting by a care provider or health care system. The continued lower volume of diagnosed breast cancer cases and screening mammograms into 2021 is concerning.

### Strengths and limitations

Institutional level radiology utilization data provided timing of changes in mammography screening. Our institution’s mammography utilization data is not linked to the patient level data used in the registry cohort. For the cohort, screening mammography method of detection is obtained from physician chart notes. The level of detailed data collected on each patient in the registry database allowed conduct of a comprehensive evaluation of multiple related impacts of delayed and restricted access to care during the pandemic, not available in larger national databases of utilization or insurance claims data. Even though the number of cases in our study is not as large as a national database, the full extent of diagnosis, breast cancer characteristics, and treatment available in the registry enabled a more complete picture of COVID-19 pandemic breast cancer care.

## Conclusions

Future tracking of breast cancer cases with treatment and diagnosis changes may be useful for guidelines in cases of natural disaster if triage of cases becomes essential again. Important lessons from the COVID-19 pandemic effect are the creation of essential surgery definitions, alternative therapy for surgical delays, and accelerated return to routine screening to remove backlogs of screening. [40] Return to previous levels of screening mammography will require population-based patient reconnection to usual care providers and related systems with scheduled reminder systems as well as return to previous staffing levels and care availability.

## Data Availability

The data underlying this article cannot be shared publicly owing to the need to protect the privacy of the study participants.
